# Medical trainees’ experiences and perceptions towards elective period; a cross sectional study

**DOI:** 10.11604/pamj.2014.17.290.4063

**Published:** 2014-04-16

**Authors:** Henry Nyongesa, Winstar Mokua, Jacob Adegu

**Affiliations:** 1 University of Nairobi, School of Medicine, Nairobi, Kenya

**Keywords:** Medical students, electives, experiences, challenges, recommendations

## Abstract

**Introduction:**

Medical electives make significant contribution in the training of medical students on healthcare outside their affiliated academic institutions. During this period, learners get exposed to different healthcare systems, diagnostic, medical and surgical techniques as well as appreciate existing challenges.

**Objective:**

To assess experiences encountered by medical students during their electives.

**Methods:**

A cross sectional study was commissioned among level 5 medical students of University of Nairobi in July 2013. A random sample of 125 students was invited to fill in self structured questionnaires after obtaining informed consent. The questionnaire which had initially been pretested on 25 nursing students comprised bio data, place, duration, funding, experiences and challenges of electives. Data obtained was analyzed using SPSS 20 and computed in terms of frequencies and percentages.

**Results:**

There were 76 (60.8% response rate) respondents with majority being males and staying in university hostel. Most of them had undertaken electives in Africa which were organized by themselves and funded mostly by their parents. There was overall positive attitude towards electives with majority (80.3%) claiming it to provide all rounded training. However, financial, transport and language barrier were identified as the main challenges (41.1%, 18.8% and 14.3%, respectively). There was a call by more than 80% of respondents for the university to collaborate with host institutions and provide stipend to cover living expenses mostly.

**Conclusion:**

The acquisition of practical skills involved in the treatment of patients forms the basis for high expectation of electives among medical trainees. It is evident that most of the participants would desire the scaling up of entire elective period through institutional collaborations and logistical support.

## Introduction

Training of a medical student into a competent and skilled healthcare provider is a long gradual process that encompasses variety of techniques. These techniques vary depending on institutional capacity, response to curricular demands and government regulatory policy, among other reasons. Generally, classroom lectures and clinical rounds in the teaching hospital is a conventional approach that is replicated across most training institutions. However to expose the student to the health system outside a training institution is one of the ways that has been pursued.

Due to the globalization of healthcare and increased migration, there has been an interest in elective periods in the training of a medical student[1]. Many institutions run this program under various names: external program, student exchange, outreach program or international health education. Although most programs are usually collaborative, there are some that are stand alone with the student seeking a place.

The importance of having such a program is for the students to be exposed to the real world situation in terms of medical practice. An acquisition of new skills and perfection of the already acquired skills is usually achieved through such a period[2]. In cross border electives, the trainees get exposed to the new technologies and different medical healthcare systems[3]. Those posted to the rural settings have an on hand experience with the challenges that face the local communities[4]. Through such avenues, the students get to learn how the communities tackle such problems in the local setting.

However noble the program is, there are a myriad of challenges that the students face. Firstly, those travelling aware from their regions are more likely to be exposed to blood borne diseases which they have not developed sufficient immunity to[5]. Sometimes, wherever the host institution is there might be a flare up of internal strife, humanitarian disaster or natural calamity. There is also a cultural identity crisis that may impede the acquisition of knowledge or interaction with the local community[6]. In light of these challenges, there is a feeling within some academic circles, that this process should be done away with[7].

Within the local setting, the university has initiated a program dubbed “PRIME-K” for posting students for medical electives in several hospitals within the country. This pioneer program caters for about 80 students who are chosen competitively. Frequently, students who miss out of the program have to organize electives out of their own volition or parental connections. Some of them forego the whole elective period due to inability to find an elective placement or fund the whole period.

Attitudes and experiences obtained during elective period have been scarcely documented. The PRIME-K documentation is largely a monitoring and evaluation process for sustainable funding of the initiative. With this a significant number of students not covered by the program yet undertook electives are missed out. Therefore we undertook the current study to assess the students’ experiences, perceptions and recommendations for the program. We believed that this will inform the policy makers on the direction of future electives.

## Methods

### Study design

A cross-sectional survey was conducted at University of Nairobi medical school in July 2013. Prior to the study, ethical clearance was sought from UoN/KNH Ethical Review Board.

### Subjects

One hundred and twenty five fifth year medical students who had undertaken electives the previous year were randomly selected and invited to fill in the questionnaire. There are usually more than 250 students who go for electives each year. To maximize response rate, a verbal request and a clear explanation on the import of the study were made immediately before the start or end of the lecture, whichever was more convenient. This exercise was performed at one sitting to minimize bias introduced by the internet or other information sources. Participants were informed that their participation in the study was anonymous and on voluntary basis. No incentives were offered. A separate informed consent form was not offered for signing since the questionnaire had instructions that filling it meant consenting to the study protocol. An exclusion criterion was based on the absence during the study.

### Questionnaire design

After literature review, a questionnaire was designed to elicit relevant data. It covered three domains: demographic variables, experiences and perceived recommendations. The initial questionnaire had been pretested on a convenience sample of 25nursing students. Questions and data findings from the pilot study were analyzed through a plenary discussion to determine if revisions to the questionnaire were necessary.

Demographic variables included in the questionnaire were gender, age stratum, study level, residence and religion. In this category, we also included place of electives whether national or international, duration and institution where the elective was done. This was followed by assessment of any challenges or benefits obtained. Several statements on recommendations were then presented, in which the subject was to respond either yes or no. Lastly, a question was asked about what respondents would prefer being funded.

### Statistical analysis

Descriptive statistics expressed as sums and percentages of all variables across all participants were computed. Statistical analysis was performed using Statistics Package for Social Sciences (SPSS) version 20.0 for windows. Analyzed data was presented in tables and charts.

## Results

The demographic profile of our respondents is shown in Table 1. The number of students who completed and returned the questionnaire were 76 representing 60.8%. Generally, there were more males than females (53.9%vs 46.1%) with all of them aged between 20-29 years. More than 50% of the 125 stayed in university hostels, with the rest staying either in private hostels or commuting from home. Christianity was the predominant religion at 81% while Hinduism was at 1.3%. Sixty eight students undertook their electives in Africa representing 89.5% while only 1 student did elective in Asia. Nationally, a majority of students 31(40.8%) did their electives in Nairobi with only 6 (7.8 %) of the participants undertaking electives in North Eastern and Eastern provinces.

**Table 1:** Demographic characteristics of respondents (N=76)

Aspect	Variable	Frequency	Percent
**Gender**	Female	35	46.1
	Male	41	53.9
			
**Age**	20-29	76	100
			
**Residence**	University hostels	39	51.3
	Private hostels	27	35.5
	Home	10	13.2
			
**Religion**	Christian	62	81.6
	Muslim	11	14.5
	Hinduism	1	1.3
	Others	2	2.6
			
**Place of electives**			
**International**	Africa	68	89.5
	Europe	4	5.3
	Americas	3	3.9
	Asia	1	1.3
			
**National**	Nairobi	31	40.8
	Central	8	10.5
	Coast	11	14.5
	Western	6	7.9
	Rift valley	8	10.5
	North eastern	3	3.9
	Eastern	3	3.9
	Nyanza	6	7.9
			
**Duration**	Less than 5 wks	9	11.8
	6-8	56	73.7
	9-11	6	7.9
	More than 11 wks	5	6.6
			
**Institution**	Academics	5	6.6
	Research	15	19.7
	Hospital	56	73.7

**Figure 1:**
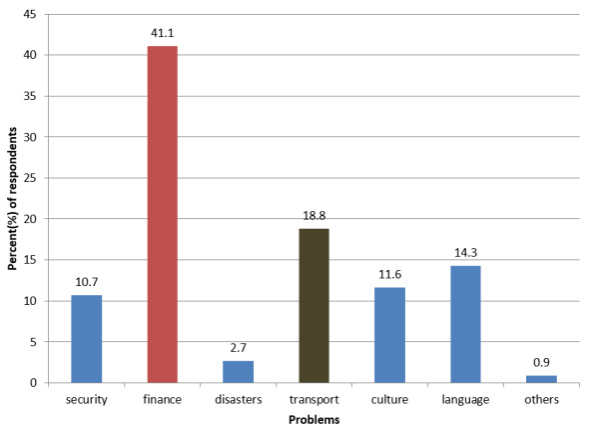
Problems encountered during electives. (N=76)

Majority of the respondents (73.7%) undertook their electives for a period of 6-8 weeks. Only 11.8% of respondents did it for less than 5 weeks and 6.6 % did for more than 11 weeks. The institution where they undertook electives was mostly a non teaching hospital 56 (73.7%) with some undertaking in academic and research institutions. Figure 1 illustrates problems encountered during the elective period. Financial challenges were chosen as the commonest problem followed by transport and language barrier at 18.8%, 14.3%, respectively.

**Figure 2:**
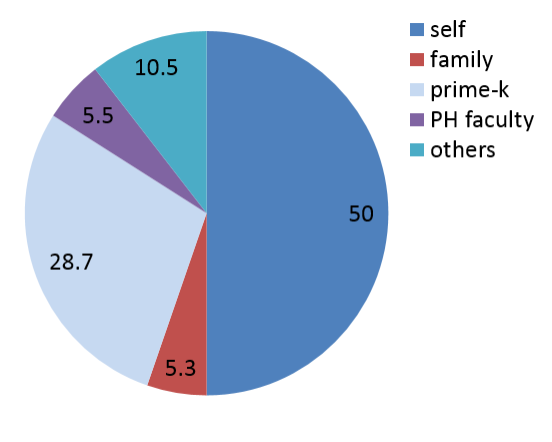
Who organized for your electives? (N= 76)

**Figure 3:**
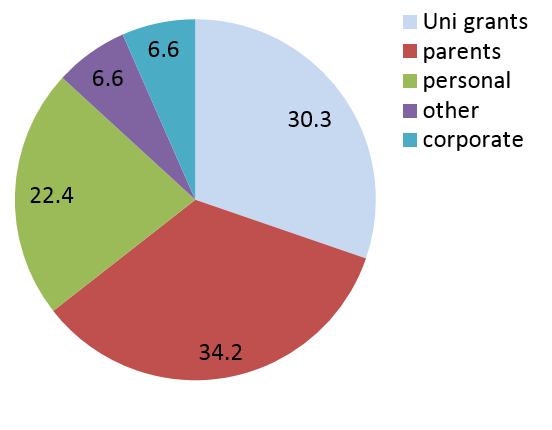
Source of funding during electives. (N= 76)

In terms of who organized for the elective program, half of the respondents did it themselves while university programs contributed to 35% of the chances (Figure 2). Most of them were funded by their parents (34.2%) while university grants catered for 30% of the respondents (Figure 3).

For those who were in hospitals, 93.4% of the respondents assisted in clinical work (Table 2). When asked if they were requested to do a procedure they did not know, 57.9% said yes with most of them (89.4%) acknowledging inadequacy. Only 7 (9.2%) reported not being supervised. Importantly, 61(80.3%) consider electives as offering all rounded training, with more than half of the respondents being influenced by the experience to choose their future specialty.

**Table 2:** Assessment of experiences and recommendations (N=76)

Question	Yes	No
5. Did you assist in clinical work during your rotation?	71(93.4)	5(6.6)
6. Were you asked to perform a procedure in which you were not competent?	44(57.9)	31(40.8)
7. Did you acknowledge your limits when asked to perform an unfamiliar	68(88.4)	8(10.5)
8. Were you being supervised during the elective period?	66(86.8)	7(9.2)
9. Do you consider elective as providing well rounded training?	61(80.3)	7(9.2)
10. Did you give any feedback to your sponsoring/learning institution	47(61.8)	28(36.8)
11. Do you think the experience will influence your choice of specialty in future?	54(71)	13(17.2)
**12. Recommendations**		
The university partner with tour host institution for sustainability.	68(89.5)	8(10.5)
Financial support is offered during electives.	69(90.8)	7(9.2)
Electives be made a must for all students.	53(69.7)	23(30.3)
Number of electives during medical training be increased	60(78.9)	16(21.1)
Formation of active organization for facilitating exposure and sponsorships	68(89.5)	8(10.5)
Electives in underserved communities i.e. slums, rural, prison	66(86.8)	10(13.1)

Almost two thirds of respondents provided feedback to the sponsoring or the learning institution. On recommendations, more than 80% of the 76 respondents proposed that the university should partner with the host institution for sustainability. Additionally, they suggested financial stipends, formation of active body mandated to facilitate such programs and doing electives in underserved community. When asked between transport and living expenses, which should be funded, most of the respondents (66.3%) chose living expenses.

## Discussion

The current study was undertaken to find out medical trainees’ experiences during their elective period. Among the key findings were that a majority of them found the elective period as providing well rounded up training. This could probably be due to hands on clinical experience, acquisition of new skills and learning of diagnostic techniques. These findings compare favourably with other studies exploring the role of electives [8–12]. We hypothesized that due to the well rounded up training, most of the undergraduates were able to identify their future specialties which is consistent with a study by Ramsey et al[13].

A majority of the participants who were asked to perform a procedure that they were not familiar with acknowledged their incompetence. This finding is consistent with Elit et al [6]. The incompetency observed could be attributed to limited exposure to clinical procedures or technological differences.

The key challenges cited by the trainees were financial, transport and language barrier. With regard to financial constraints, most of the participants were relying on their parents’ funds. The parents could have been financially incapable due to competing interests like clearance of school fees. Information concerning electives usually comes after release of results hence parents could also not have had time to factor in this. This could have lead to most students recommending the university to finance the program. Since most of the participants are conversant in two languages only: English and Kiswahili, they might have encountered communication barriers with people in far flung areas. In some studies conducted particularly in the West, these findings have been noted[14, 15]. Subjects reported difficulty in talking to the rural folks. In some areas, there were inadequate or absent means of transport from their areas of residence to the hospital.

For sustainability, efficiency and accountability of the program, the participants recommended that the university partners with the institutions that they had undertaken electives in. They equally suggested that electives should be conducted also in underserved communities. The logic behind the recommendation was probably due to the thought that they present unique health challenges and the urge to practice medicine in a diverse setting as deciphered from some studies [4, 16].

This study should be interpreted in light of some limitations. This being a cross sectional study, we could not attribute the causality to the findings obtained. Secondly, due to the uniqueness of the institution's curriculum, the results in the current study may not be generalized to other institutions especially outside the country. However, we believe that this being a pioneer institution in the country, the results may be replicated across local institutions since they derive almost same approach to training of undergraduates. To the best of our knowledge, this is the first study of its kind hence it could act as a bench mark for elective policy formulation.

## Conclusion

In conclusion, medical trainees have a high expectation of electives. They see it as an opportunity to get hands on clinical skills and get directly involved in the management of patients. It is apparent that most of the participants would wish for scaling up of entire elective period through institutional collaborations and logistical support.
